# Antipsychotic monotherapy and polypharmacy in the naturalistic treatment of schizophrenia with atypical antipsychotics

**DOI:** 10.1186/1471-244X-5-26

**Published:** 2005-05-27

**Authors:** Douglas Faries, Haya Ascher-Svanum, Baojin Zhu, Christoph Correll, John Kane

**Affiliations:** 1 Outcomes Research, Eli Lilly and Company, Indianapolis, Indiana, USA; 2 Department of Psychiatry, The Zucker Hillside Hospital, Glen Oaks, New York, USA

## Abstract

**Background:**

Antipsychotic monotherapy is recognized as the treatment of choice for patients with schizophrenia. Simultaneous treatment with multiple antipsychotics (polypharmacy) is suggested by some expert consensus guidelines as the last resort after exhausting monotherapy alternatives. This study assessed the annual rate and duration of antipsychotic monotherapy and its inverse, antipsychotic polypharmacy, among schizophrenia patients initiated on commonly used atypical antipsychotic medications.

**Methods:**

Data were drawn from a large prospective naturalistic study of patients treated for schizophrenia-spectrum disorders, conducted 7/1997–9/2003. Analyses focused on patients (N = 796) who were initiated during the study on olanzapine (N = 405), quetiapine (N = 115), or risperidone (N = 276). The percentage of patients with monotherapy on the index antipsychotic over the 1-year post initiation, and the cumulative number of days on monotherapy were calculated for all patients and for each of the 3 atypical antipsychotic treatment groups. Analyses employed repeated measures generalized linear models and non-parametric bootstrap re-sampling, controlling for patient characteristics.

**Results:**

During the 1-year period, only a third (35.7%) of the patients were treated predominately with monotherapy (>300 days). Most patients (57.7%) had at least one prolonged period of antipsychotic polypharmacy (>60 consecutive days). Patients averaged 195.5 days on monotherapy, 155.7 days on polypharmacy, and 13.9 days without antipsychotic therapy. Olanzapine-initiated patients were significantly more likely to be on monotherapy with the initiating antipsychotic during the 1-year post initiation compared to risperidone (p = .043) or quetiapine (p = .002). The number of monotherapy days was significantly greater for olanzapine than quetiapine (p < .001), but not for olanzapine versus risperidone, or for risperidone versus quetiapine-initiated patients.

**Conclusion:**

Despite guidelines recommending the use of polypharmacy only as a last resort, the use of antipsychotic polypharmacy for prolonged periods is very common during the treatment of schizophrenia patients in usual care settings. In addition, in this non-randomized naturalistic observational study, the most commonly used atypical antipsychotics significantly differed on the rate and duration of antipsychotic monotherapy. Reasons for and the impact of the predominant use of polypharmacy will require further study.

## Background

Guidelines for treating patients with schizophrenia [1-5] have long recognized antipsychotics as the core treatment modality and have consistently recommended antipsychotic monotherapy as the treatment of choice. Although expert consensus guidelines do not advocate antipsychotic polypharmacy, some [4] suggest antipsychotic polypharmacy as the last resort after having exhausted prior monotherapy alternatives. Only one of the consensus guidelines [5] offers guidance on the duration of antipsychotic polypharmacy, which is recommended when switching from one antipsychotic to another (cross titration or overlap and taper) and for not longer than 60 days.

Monotherapy is recognized as the preferred mode of treatment because it allows clinicians to accurately evaluate the patient's response to a new course of treatment [6]. Monotherapy permits documenting patient's response to an adequate trial of each medication, helping to reduce the complexity of the medication regimen, reducing the risk of adverse events, and making it easier to assess and manage future symptom exacerbations [7].

Despite consistent recommendations of antipsychotic monotherapy, polypharmacy is widespread in the treatment of schizophrenia [7-15]. The proliferation of antipsychotic polypharmacy is likely driven by increased availability of pharmacologically diverse atypical antipsychotics that augment an extensive armamentarium of typical antipsychotics. Generally, the concurrent use of more than one antipsychotic, particularly of typical and atypical agents, was reported to vary from 13% to 60%, depending on the population studied, the year when the study has been conducted, the study method, the type of treatment site, and the duration of the study period [8,9,14,16-19].

The body of evidence supporting the benefits of antipsychotic polypharmacy is limited [7,8,20,21] and is in contrast to the extensive and compelling body of evidence supporting monotherapy with atypical antipsychotics [6,7,9,11-13,22-24].

Antipsychotic polypharmacy was reported to increase the risk of medication-related adverse events and of drug-drug interactions, to increase the need for additional medications to treat emerging side-effects, to decrease adherence with medication due to increased treatment complexity, confounding clinicians' ability to discern helpful from unhelpful medications, and to increase cost of care [7,10,25]. A recent case-control study of psychiatric inpatients demonstrated that short-term treatment with antipsychotic polytherapy was associated with major increases in drug exposure, in adverse events, and in hospitalized duration but with no apparent clinical benefit [16].

Antipsychotic polypharmacy appears to be used for various reasons [7,11], with the one cited most often being the wish to bolster medication effectiveness in treating patients with refractory psychotic symptoms, mood symptoms, or behavioral problems [9]. It is, however, unclear if antipsychotic polypharmacy is associated with specific atypical antipsychotics more often than with others.

The body of research on differential monotherapy or polypharmacy among atypical agents is sparse [8,26-30]. It is unclear if risperidone and quetiapine-treated patients differ on monotherapy parameters. Most previous studies reported higher monotherapy or lower polypharmacy rates for olanzapine compared to risperidone [18,19,28,29], and compared to quetiapine-treated patients [25,27,30], whereas olanzapine and risperidone-treated patients were not found to significantly differ on polypharmacy rates in three studies [25-27]. The reasons for the inconsistent findings are unclear but may stem from methodological issues, including lack of complete information about the use of depot antipsychotics, about use of medications during psychiatric hospitalizations, and inadequate control for potential selection effects, which arise from clinicians' tendency to tailor treatment regimens to patients' illness profiles and previous treatment patterns.

This study expanded on prior research by using comprehensive medication data from a large prospective multi-site naturalistic study of patients treated for schizophrenia-spectrum disorders in the United States to assess the annual rate and duration of monotherapy, and its inverse, antipsychotic polypharmacy. We also focused on patients initiated on commonly used atypical antipsychotics – olanzapine, quetiapine, or risperidone – and compared their rates and duration of monotherapy during the year following initiation on the antipsychotic medication.

## Methods

### Data source

This study used data of the U.S. Schizophrenia Care and Assessment Program (US-SCAP), a large (N = 2327) non-randomized, naturalistic, 3-year prospective multi-site study conducted between 7/1997 and 9/2003. The goal of US-SCAP was to understand the treatment of patients with schizophrenia in usual care settings. Approximately 400 patients at each of the study's six regional sites were enrolled. All participants were diagnosed with schizophrenia, schizoaffective, or schizophreniform disorders based on DSM-IV criteria, and were at least 18 years of age. Patients were excluded if they were unable to provide informed consent or had participated in a clinical drug trial within 30 days prior to enrollment.

In order to reduce selection bias, outpatients were randomly selected from site medical information rosters of active clients. Inpatients were sequential admissions. Of 3332 patients who met inclusion criteria, 765 (23.0%) refused, and 240 (7.2%) were not enrolled for other reasons. Most enrollees competed 1 year of follow-up (78.1%), with fewer participants completing 2 years (69.6%), and 3 years (65.2%). Of the 2327 enrollees, 21.2% were hospitalized at enrollment or during the 6 months prior to enrollment, and 36.2% were hospitalized in the year prior to enrollment.

Participants were enrolled from six states (California, Colorado, Connecticut, Florida, Maryland, and North Carolina) and represented treatment in diverse systems of care including community mental health centers, university health care systems, the Department of Veterans Affairs Health Services (VA), and community and state hospitals. Institutional Review Board (IRB) approval was received at each regional site and informed consent was received from all participants.

At enrollment, almost all participants (94.7%) were treated with at least one antipsychotic medication, including oral typical (36.7%), oral atypical (58.1%), and depot typical antipsychotics (19.6%). Medication changes during the study period, including initiations and discontinuations, if any, were based on physicians' decisions as they occur in usual care. Further details about US-SCAP are available elsewhere [31,32].

The current study included data of US-SCAP participants who were initiated on one of three commonly used atypical antipsychotics – olanzapine, quetiapine, or risperidone, in regular oral formulation. Participants were defined as initiators if they were not prescribed the index antipsychotic for at least 60 days prior to initiation, had at least 2 months of treatment data available prior to initiation of the index antipsychotic, and had at least 12 months of follow-up information in the study post initiation. Inclusion of data for the 2 months prior to initiation allowed for identification of variables on which the treatment groups differed at the time of initiation. Further, because this study aimed to assess use of antipsychotics over a 1-year period, availability of a 12-month follow-up period was necessary. Note that patients were not excluded from the analysis if they stopped their antipsychotic therapy – only if they discontinued or completed the study within less than 12 months post initiation. Some patients may have met inclusion criteria for being an initiator of more than one medication throughout the 3-year period. Such patients were considered to be initiators of the first antipsychotic medication they initiated in the study.

### Outcome measures

Medical records provided information about prescribed psychiatric medications and were systematically abstracted for the 6 months prior to enrollment and for each 6-month interval thereafter. Patients were queried about the use of medications and other mental health resources outside those received at their regular treatment site. When this occurred, systematic efforts were made to abstract out-of-site medical records.

On a daily basis, monotherapy (polypharmacy) was defined as the occurrence of one (more than one) ongoing antipsychotic medication prescription. Predominant use of monotherapy (polypharmacy) was defined as the use of monotherapy (polypharmacy) for > 300 days out of the year. Substantial monotherapy (polypharmacy) use was defined as the use of monotherapy (polypharmacy) for > 60 to ≤ 300 days out of the year. Consistent with expert consensus guidelines [5], prolonged polypharmacy was defined as a period of more than 60 *consecutive *days of polypharmacy.

Two parameters of antipsychotic monotherapy were used as the outcome measures for initiating treatment group comparisons (olanzapine, quetiapine, or risperidone): (a) the percentage of patients on antipsychotic monotherapy with the index atypical antipsychotic over the 1-year post initiation, and (b) the cumulative number of days of antipsychotic monotherapy.

Participants were assessed with standard psychiatric measures at enrollment and at 12-month intervals thereafter. These measures were not administered, however, at the time of initiation or discontinuation of any medication. Consequently, these variables were not used in this study. Furthermore, the study did not assess reasons for medication initiation or discontinuation, thus eliminating the ability to evaluate the reasons for any medication changes.

### Statistical methods

Summary statistics were used to quantify monotherapy/polypharmacy use at initiation on the medication and across the 1-year period post initiation for all patients (N = 796). This included summarizing the distribution of durations on monotherapy/polypharmacy as well as classification of patients based on predominant, substantial monotherapy/polypharmacy definitions and the prolonged use of polypharmacy.

To assess differences in the use of monotherapy between the treatment groups, two statistical approaches were utilized. First, treatment group differences in the percentage of patients on monotherapy each day over the 1-year following initiation were assessed using a repeated measures general linear model (GEE, Generalized Estimating Equations) [33] with an exchangeable correlation matrix. GEE models are widely used for analyzing longitudinal binary data as they provide consistent parameter estimates and account for the correlation of responses within patients over time. The model consisted of terms for treatment, time, treatment by time interaction, and a set of a priori determined covariates discussed below. Second, treatment group differences in the mean number of days of monotherapy over the 1-year following initiation were assessed. As the distribution of the number of days was non-normal – with a high percentage of patients with zero days, a non-parametric propensity score adjusted bootstrap re-sampling approach was utilized [34].

In all treatment comparisons, differences were adjusted for a set of available covariates selected *a priori*. These covariates were selected based on the expectations that they may be associated with monotherapy use. Some clinical variables that were collected at 1-year intervals in this study were not used as covariates because they were not collected at the point of initiation or discontinuation of any medication. Thus, the covariates used for adjustment in this study included available socio-demographic and medication history variables: age, gender, ethnicity, illness duration, monotherapy status at initiation (yes/no), investigational site, time, time by treatment interaction, and the following variables based on the 2 months prior to initiation of the index antipsychotic: any psychiatric hospitalization, number of days of antipsychotic monotherapy, and any use of: typical antipsychotics, atypical antipsychotics, antiparkinsonian agents, antidepressants, anti-anxiety medications, sleep agents, mood stabilizers, and typical antipsychotics in depot formulation.

No adjustment for multiple comparisons was performed as we considered the repeated measures GEE model the primary analysis and the monotherapy duration analysis as a secondary analysis to assess the robustness of the primary results.

## Results

### Patient characteristics

Table 1 summarizes the socio-demographic characteristics and medication history for the patient population. The sample (N = 796) included patients who were initiated during the study on olanzapine (N = 405), quetiapine (N = 115), or risperidone (N = 276). While similar in many aspects, the treatment groups differed on several characteristics – most notably the percentage of patients on antipsychotic polypharmacy at initiation, which was highest among quetiapine-treated patients and lowest for the risperidone treatment group. During the 2 months prior to initiation on the index antipsychotic, a greater percentage of quetiapine-treated patients were treated with atypical antipsychotics and mood stabilizers; a greater percentage of olanzapine-treated patients were treated with typical antipsychotics in both oral and depot formulations; risperidone patients had fewer antipsychotic monotherapy days. Further, the quetiapine treatment group had least males, the lowest illness duration, and fewest patients with substance use disorders.

**Table 1:** Baseline characteristics of patients initiated on olanzapine, risperidone, and quetiapine

**Variable**	**Olanzapine N = 405**	**Quetiapine N = 115**	**Risperidone N = 276**
Age, mean (S.D.)	41.8 (10.5)	39.6 (10.9)	40.4 (12.1)
Illness duration (yrs), mean (S.D.) * ^a,b^	22.1 (11.3)	18.5 (11.2)	19.9 (12.4)
Male gender* ^b^	61.7%	47.8%	54.7%
Ethnicity			
White	46.7%	53.9%	45.3%
Black	41.2%	33.9%	36.6%
Other	12.1%	12.2%	18.1%
Schizoaffective disorder	32.6%	40.0%	33.3%
Substance-use disorder* ^b,c^	30.9%	19.3%	29.4%
No insurance	6.9%	8.9%	9.2%
Polypharmacy at initiation * ^a,c^	67.2%	76.5%	59.8%
Prior treatment pattern †			
Prior Antipsychotic monotherapy days, mean (S.D.)* ^a^	42.4 (26.0)	39.2 (27.7)	36.8 (28.0)
Prior psychiatric hospitalization* ^a^	21.0%	21.7%	29.4%
Prior atypical antipsychotic* ^b,c^	23.5%	69.6%	28.6%
Prior typical oral antipsychotic* ^b,c^	63.5%	39.1%	58.7%
Prior typical depot antipsychotic* ^a,b^	23.2%	13.9%	15.9%
Prior antidepressants	35.6%	45.2%	40.9%
Prior anti-anxiety agents	10.4%	14.8%	12.0%
Prior antiparkinsonians agents	46.9%	36.5%	47.5%
Prior mood stabilizers* ^a,c^	30.1%	39.1%	23.2%
Prior sleep agents	0.7%	3.5%	2.2%

* Significant groups difference at p < 0.05

^a^significant pairwise comparisons olanzapine vs. risperidone

^b ^significant pairwise comparisons olanzapine vs. quetiapine

^c ^significant pairwise comparisons risperidone vs. quetiapine

† Treatment pattern during the 2 months prior to initiation on the index drug

### Medication dose

The dosing of each antipsychotic during the year post initiation, at initiation and at endpoint, and by monotherapy versus polypharmacy status at initiation is presented in Table 2. Mean doses were higher at endpoint than at initiation and were within the package insert guidelines for the vast majority of the patients. In addition, the mean doses were higher for those on monotherapy than polypharmacy at initiation, but this was not always found at endpoint.

**Table 2:** Daily Dose (mg/day) of Index Atypical Antipsychotic: During the 1-Year Post Initiation, at Initiation, and at Endpoint

**Group/Time**	**Mean (SD)**	**Median**	**Min/Max**	Mean Mono/Poly*
Olanzapine				
1-Year	13.9 (7.4)	10.0	2.5/40	15.1/13.5
Initiation	10.0 (6.1)	10.0	2.5 / 40	10.7 / 9.5
Endpoint	14.2 (8.4)	10.0	2.5 / 50	13.9 / 14.5
				
Quetiapine				
1-Year	330 (214)	295	25/850	305/334
Initiation	164.2 (151)	100	12.5 / 800	223 / 138
Endpoint	341 (277)	300	25 / 1300	369 / 330
				
Risperidone				
1-Year	4.2 (2.3)	3.9	0.28/12.9	4.7/3.9
Initiation	2.7 (2.0)	2.0	0.25 / 12	3.2 / 2.4
Endpoint	4.3 (2.7)	4.0	0.25 / 16	4.5 / 4.1

* Mean Mono/Poly indicates the mean dose (mg/day) for patients while on antipsychotic monotherapy, and while on antipsychotic polypharmacy

### Overall rates and duration of antipsychotic monotherapy/polypharmacy

Approximately one-third (34%) of the 796 patients were initiated on antipsychotic monotherapy with the index antipsychotic. Conversely, most patients (66%) received another antipsychotic, or polypharmacy, at the time of initiation. Only 30% of the polypharmacy-initiated patients were deemed to be in the process of medication change because their polypharmacy ceased within 60 days following initiation on the atypical agent.

**Figure 1:**
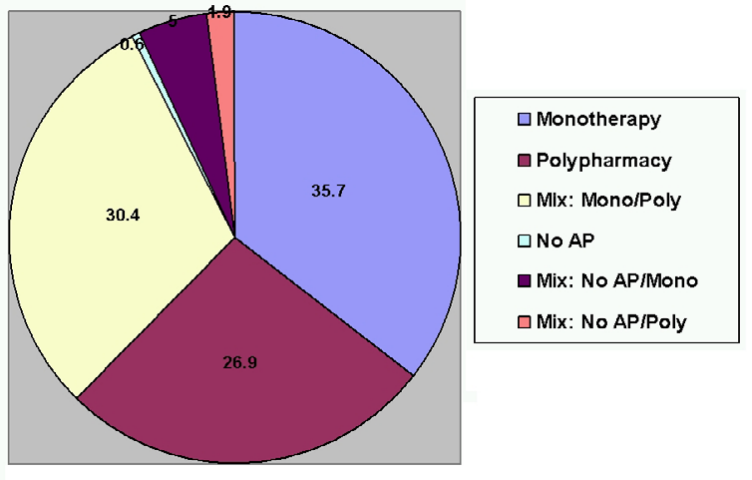
Percent of patients in each monotherapy/polypharmacy treatment category. Abbreviations: Mono, monotherapy; Poly, polytherapy; No AP, no antipsychotic treatment. Definitions: Monotherapy: Predominant antipsychotic monotherapy (> 300 days); Polypharmacy: Predominant antipsychotic polypharmacy (> 300 days); Mix: Mono/Poly: Substantial periods of monotherapy (61 to 300 days) and substantial periods of polypharmacy (61 to 300 days), but no substantial periods of no antipsychotic treatment (< 60 days); No Antipsychotic: Patients predominately without prescribed antipsychotics (> 300 days); Mix: No AP/Mono: Patients predominately without prescribed antipsychotics (> 300 days) and substantial periods of monotherapy (61 to 300 days); Mix: No AP/Poly: Patients predominately without prescribed antipsychotics (> 300 days) and substantial periods of polypharmacy (61 to 300 days).

The distribution of monotherapy and polypharmacy treatment categories during the 1-year period (Figure 1) indicates that about a third (35.7%) of the patients were treated predominately with monotherapy (>300 days), 26.9% were treated predominately with polypharmacy (>300 days), 30.2% had a mix of both substantial monotherapy and polypharmacy treatment periods (61–300 days of each), and 0.6% were not treated with any antipsychotic for more than 300 days. A small proportion of the patients (6.6%) had periods without antipsychotic medications along with substantial treatment periods with either monotherapy or polypharmacy (61–300 days).

**Figure 2:**
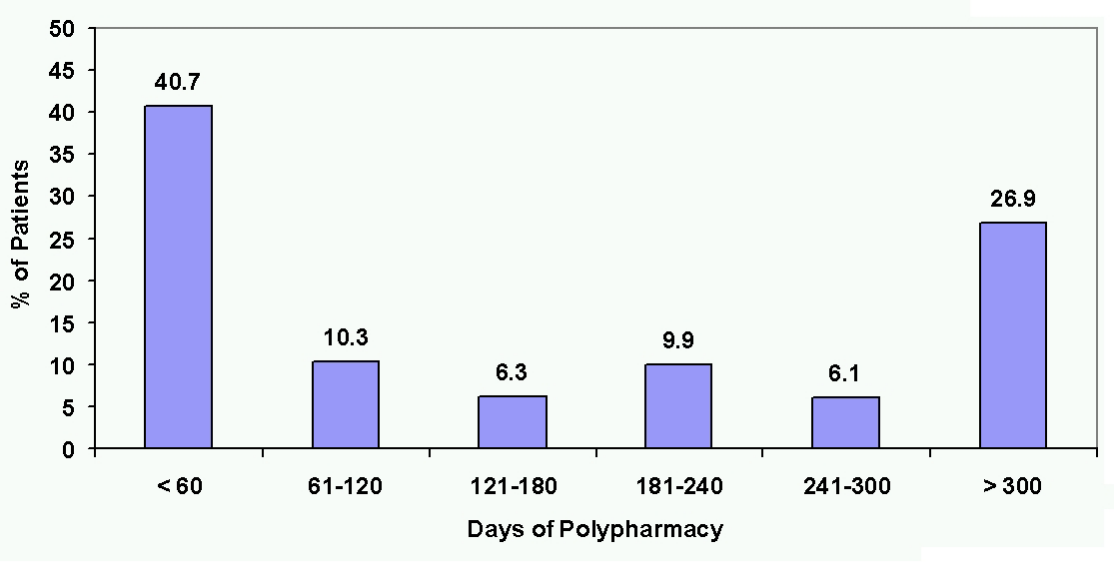
Percent of patients with antipsychotic polypharmacy during the 1-year period by duration category.

Overall, patients averaged 195.5 days on monotherapy (54% of the year), 155.7 days on polypharmacy (43% of the year), and 13.9 days without antipsychotic therapy (3% of the year). The distribution of the average number of polypharmacy days for each patient over the 1-year period (Figure 2) indicates that 40.6% had polypharmacy for less than 60 days. In fact, most patients (57.7%) had at least one prolonged period of antipsychotic polypharmacy (longer than 60 consecutive days).

### Specific rates and duration of monotherapy/polypharmacy

A significantly greater percentage of olanzapine-treated patients were on antipsychotic monotherapy during the 1-year period compared to risperidone and to quetiapine. Olanzapine-treated patients were 2.08 times more likely to be on monotherapy than quetiapine (Odds Ratio (OR) = 2.08, 95% Confidence Interval (CI), 1.30–3.31, p = .002), and 1.36 times more likely to be on monotherapy than risperidone (OR = 1.36, 95%CI, 1.01–1.84, p = .043). Differences between risperidone and quetiapine initiators showed a trend toward significance (OR = 1.53, 95% CI, 0.94–2.47, p = .085).

**Figure 3:**
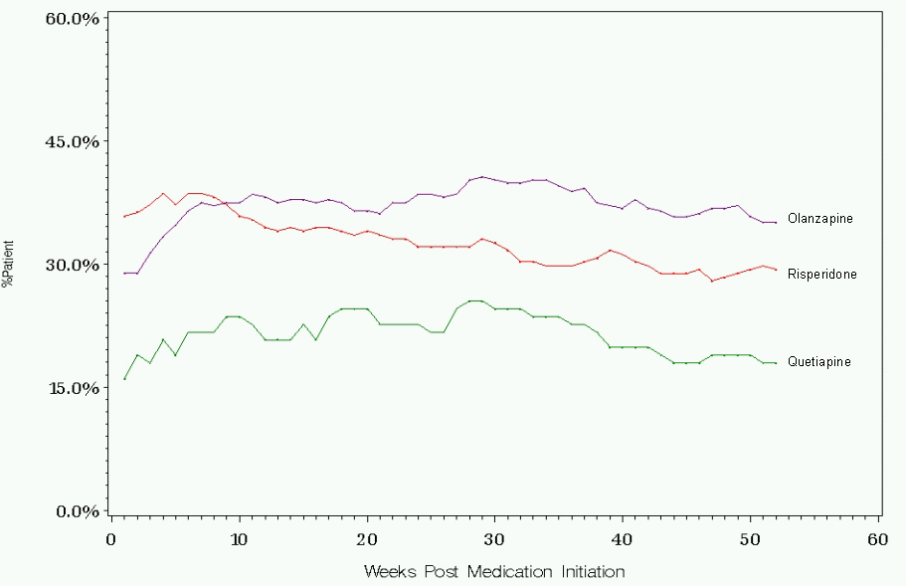
Percent of patients on monotherapy during the 1-year following initiation on the atypical antipsychotic medication. Using a GEE (generalized estimating equations) repeated measures binary data model, pair-wise comparisons found a significantly higher rate of monotherapy for olanzapine compared to quetiapine (p = .002) and risperidone (p = .043). The difference between risperidone and quetiapine approached statistical significance (p = .085).

**Figure 4:**
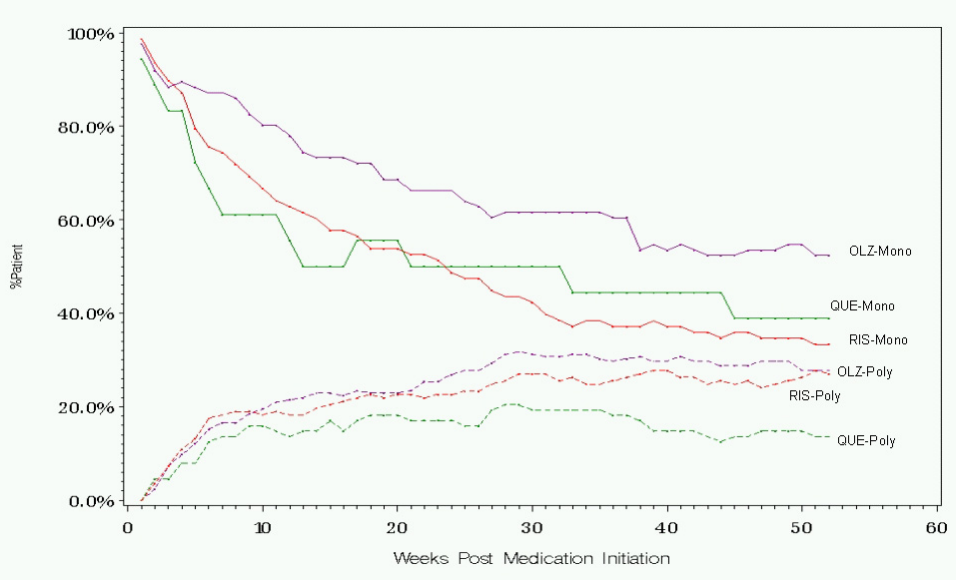
Percent of patients on monotherapy after initiation on antipsychotic polypharmacy or antipsychotic monotherapy. Abbreviations: OLZ, olanzapine; Poly, polypharmacy at initiation; QUE, quetiapine; RIS, risperidone; Mono, monotherapy at initiation

The percentages of patients on antipsychotic monotherapy during every week in the 1-year following initiation for each treatment group are provided in Figure 3. Due to the strong influence of monotherapy status at initiation on later monotherapy status, and the treatment differences in monotherapy status at initiation, results are also shown stratified by monotherapy status at initiation in Figure 4.

The observed mean number of monotherapy days on the initiating antipsychotic for olanzapine, quetiapine, and risperidone are presented in Table 3 for patients initiating on monotherapy and for patients initiating on polypharmacy. For monotherapy or polypharmacy initiators, patients on olanzapine had a greater number of days of monotherapy on the initiating medication while quetiapine initiators had the fewest. Using a propensity score bootstrap analysis, pair-wise comparisons found a significantly longer duration on monotherapy for olanzapine compared to quetiapine (p < .001) and a trend toward longer duration for risperidone initiators as compared to quetiapine (p = .052). The difference between olanzapine and risperidone was not statistically significant.

**Table 3:** Number of monotherapy days on the initiating antipsychotic during the 1-year post initiation

	**Status at Initiation**				
**Treatment**	**Monotherapy**		**Polypharmacy**		**Overall Mean**
	N	Mean (SD)	N	Mean (SD)	(SD)
Olanzapine	133	252.1 (132.8)	272	90.2 (126.8)	143.4 (149.5)
Quetiapine	27	159.8 (142.4)	88	53.6 (102.1)	78.5 (120.9)
Risperidone	111	230.6 (143.8)	165	78.0 (121.2)	139.3 (150.5)

Abbreviation: SD, standard deviation.

## Discussion

In this large prospective naturalistic study of patients with schizophrenia, antipsychotic polypharmacy was found to be highly prevalent and to be of prolonged duration. Overall, most patients (57.7%) had at least one period of antipsychotic polypharmacy longer than 60 consecutive days, and only a third (35.7%) of the patients were treated predominately with monotherapy. More specifically, two thirds (66%) of the patients were treated with another antipsychotic at the time of initiation on the atypical agent, a practice that could have signaled a medication change process. However, most of these patients continued on antipsychotic polypharmacy for a substantial duration and only a small proportion of those patients (30%) were deemed to have gone through medication changes as polypharmacy ceased within the first 60 days after medication initiation. Findings suggest that for the majority of patients, polypharmacy is a prolonged and deliberate treatment choice rather than an interim, brief, or unintentional practice.

A third (34%) of the patients were not receiving another antipsychotic at the time of initiation on the studied atypical medications (olanzapine, quetiapine, and risperidone). These monotherapy-initiated patients continued on monotherapy for only about a third of the year post initiation, and at the end of that year, more than 50% of them were no longer receiving monotherapy with the initiating antipsychotic. Further attesting to the pervasive practice of antipsychotic polypharmacy is the finding that over 40% of all patients had no days of monotherapy with the initiating atypical antipsychotics during the 1-year treatment period. On the average, patients were treated with monotherapy for 54% of the year, with polypharmacy for 43% of the year, and without antipsychotic therapy for 3% of the year.

While polypharmacy was found to be prevalent in this patient population, the question of generalizability remains. However, a goal in designing the US-SCAP study was to generate a sample of patients representative of those treated in usual care. Participants in this large prospective naturalistic study were treated for schizophrenia at large public health care delivery systems in the United States, and were enrolled from multiple sites across six states. The patients were randomly identified from active client rosters at each site and only then approached about enrollment in the study. In addition, there were few exclusion criteria that would restrict the patient population.

This study provided a 1-year longitudinal perspective on the rate and duration of polypharmacy following initiation on the index antipsychotic whereas most previous studies assessed polypharmacy during shorter time periods, such as a 2-month window [15], or during inpatient hospitalization [16,19,25,26]. Generally, the larger is the studied time window, the higher is the likelihood of finding polypharmacy. Another major difference is the age of the data because polypharmacy has increased over the years. Studies using data from the earlier years after the introduction of the atypical antipsychotics [10,14] tend to report lower prevalence rates of antipsychotic polypharmacy, whereas studies using more recent data reported higher polypharmacy rates [8,15]. The complexity involved in comparing findings across studies is further compounded by variations in the definition of polypharmacy. Although most studies defined polypharmacy as any time with more than one antipsychotic [15], others have set specific time requirements, such as at least 14 days of concurrent antipsychotic use [8]. Other differences in study methods can generate different results. While the current study followed patients after their initiation on certain antipsychotic medications, other studies used a cross sectional method, assessing the prevalence of polypharmacy at a given time window, without using time of initiation on the studied antipsychotic medications as the reference point. While cross sectional designs provide important information about prevailing practices at a given time window, the cross sectional method is not well suited for comparisons between antipsychotic treatment groups. When the date of initiation on the antipsychotic is not used as a starting point, treatment group differences may be obscured by differential duration on the medication prior to the studied time window, data that are not included in cross sectional designs.

This study also found significant differences in monotherapy and polypharmacy between the most commonly prescribed atypical antipsychotic medications. Patients initiated on olanzapine were significantly more likely to be on monotherapy compared to quetiapine (rate and duration) or risperidone-initiated patients (rate only) during the 1-year post treatment initiation. The current findings appear consistent with several previous studies in which olanzapine-treated patients were found to have significantly higher rate of monotherapy compared to risperidone [18,19,28,29], and compared to quetiapine-treated patients [25,27,30], with the quetiapine treatment group being the least likely to be treated monotherapy [8,27]. Findings are particularly congruent with those reported in a study of medication patterns in the Michigan Medicaid database [28], in which the proportion of schizophrenia patients treated with olanzapine monotherapy remained relatively steady over the first 3 months of treatment while the proportion of patients receiving risperidone monotherapy decreased over time.

The present study helps demonstrate the dynamic and complex nature of medication management of patients with schizophrenia in usual clinical practice [14]. In addition to providing new information on the rate of monotherapy during treatment with various atypical agents, this study further contributes to the literature by providing a longitudinal perspective on the duration of monotherapy/polypharmacy following treatment initiation. The strengths of this study appear to lie in its large representative and diverse sample, the ability to provide comparative data on a number of commonly used atypical antipsychotics, the availability of comprehensive medication information about use of antipsychotics in depot formulation and about antipsychotics used during hospitalizations (types of data often absent in claims databases), and notably, the ability to generalize the findings to patients treated at large public systems of health care across the United States.

This study also has its limitations. First is the use of naturalistic observational data to compare antipsychotic treatment groups, because observed group differences in rates or duration of monotherapy could result from pre-existing differences between the treatment groups rather than differences in medication choice. Physicians tend to select treatments for different types of patients and illness profiles, a practice that may lead to lack of comparability between the treatment groups at initiation. Analyses that do not appropriately control for such differences can be biased, and one can never be certain that all important group differences have been controlled for statistically. Indeed, in this study differences were observed between groups at initiation. Although differences in several available pre-existing patient and treatment characteristics were controlled for in the analysis, it is possible that other pre-existing group differences were present. For instance, the design of this study did not include assessment of symptom severity or substance use at the time of initiation on the index antipsychotic. Thus, differences in symptom severity or substance use at initiation could not be controlled for.

Another study limitation is lack of information about reasons for initiating antipsychotic medication changes or the specific treatment indications. This type of information was not assessed in US-SCAP but could be valuable in discerning whether there is a link between treatment effectiveness and antipsychotic monotherapy (or polypharmacy). Treatment indication is also important because some antipsychotics, particularly quetiapine in low doses, may have been used to treat insomnia rather than core symptoms of schizophrenia. Although some have speculated that higher doses of quetiapine produce better outcome, the available data [35] do not support this. Davis and Chen [35] observed, however, "the sponsor of quetiapine has 2 studies of dose response, one nearing completion, and the other just beginning, so more information will be available in the future". They also noted "the fact that individuals do clinically improve at higher doses in open studies does not prove that the higher dose was responsible, as such improvement might reflect the passage of time, augmenting drugs, or other confounding factors."

Further, this study did not control for potential "sponsorship" bias that could have influenced clinicians' prescription rates and duration of monotherapy in favor of the sponsor's antipsychotic medication (in this case, olanzapine). Although we cannot completely rule out this possibility, sponsor bias is unlikely to have played a role in this study because the current findings are highly consistent with findings of other studies conducted by independent researchers who used non-industry-sponsored data such as Medicaid claims database [18,19,28,29]. Additional support for the validity of our findings and their freedom from sponsorship bias comes from a recent double-blind randomized study conducted by the sponsors of risperidone [35], comparing risperidone and quetiapine on the rate of antipsychotic polypharmacy. That short term trial found the relative risk (quetiapine vs. risperidone) of using antipsychotic polypharmacy to be higher for quetiapine compared to risperidone (odds ratio 1.90, 95% CI, 1.29–2.80, p = 0.001), a finding similar to that in our observational study, in which the odds ratio for antipsychotic polypharmacy of quetiapine vs. risperidone was 1.53 (95% CI 0.94–2.47, p = 0.085).

This study also did not present information about concomitant use of psychotropic medications other than antipsychotics, thus providing a partial understanding of the full range of psychotropic polypharmacy that takes place in usual practice. This is a complex phenomenone that will benefit from a separate and detailed study. We, however, cognizant of the potential role of prior concomitant psychotropic medications and used five types of psychotropics (antidepressants, anti-anxiety, antiparkinsonian, mood stabilizers, and sleep agents) in the analyses. Because prior use of concomitant psychotropics is highly correlated with continued use of these concomitant medications post initiation of the new antipsychotic regimen, we opted to include patients' prior concomitant medication as covariates in the analyses. The correlations between concomitant psychotropic medication use prior to initiation on the index drug and its concomitant use post initiation on the antipsychotic were consistently high, ranging from .65 to .81 (r = .81 for antidepressants, r = .80 for anti-anxiety agents, r = .73 for antiparkinsonians, r = .81 for mood stabilizers, and r = .65 for sleep agents). These correlations suggest that the bigger factor in the use of concomitant psychotropic medications post antipsychotic initiation is likely patients' prior medication history and not the antipsychotic monotherapy/polypharmacy status. Generally, the mean daily total number of concomitant psychotropic medications was higher for patients on antipsychotic polypharmacy (1.56) than for patients on antipsychotic monotherapy (1.24). Lastly, this study did not address the potential impact of polypharmacy on the total cost of antipsychotic treatment, or on patients' clinical outcomes, an area of investigation that will require further study.

## Conclusion

This large prospective study of treatment in usual care settings demonstrated that antipsychotic medication management of schizophrenia patients is a complex process characterized by prevalent and prolonged polypharmacy. Current findings also highlight differences between the most commonly used atypical antipsychotics on the rate and duration of antipsychotic monotherapy, and its inverse, antipsychotic polypharmacy. Future research is needed to clarify the reasons for the observed treatment group differences, and to investigate the potential impact of antipsychotic polypharmacy on the costs of treatment and other important treatment outcomes in the long-term medication management of patients with schizophrenia.

## Competing interests

The Schizophrenia Care and Assessment Program (SCAP-US) was funded by Eli Lilly and Company. The article-processing charge for this article was paid by Eli Lilly and Company. Dr. Kane serves as a consultant and/or lecturer for Eli Lilly and Company, Abbot, Bristol-Meyers Squibb, Pfizer and Janssen. Dr. Correll serves as a consultant and/or lecturer for Astra Zeneca, Bristol-Meyers Squibb, Elli Lilly and Company, and Janssen. Drs. Ascher-Svanum, Faries and Zhu are employees of Eli Lilly and Company. Drs. Ascher-Svanum, Faries and Zhu are minor stock holders in Eli Lilly and Company. Drs. Kane and Correll do not hold any stocks or shares in an organization that may in any way gain or lose financially from the publication of this manuscript, either now or in the future. The authors do not hold and are not applying for any patents relating to the content of the manuscript, and have not received reimbursements, fees, funding, or salary from an organization that holds or has applied for patents relating to the content of the manuscript. The authors have no other financial competing interests, and have no non-financial competing interests.

## Authors' contributions

DF conceived of the study, designed the analytical plan, performed the statistical analyses, interpreted the results, and drafted the manuscript. HAS participated in study design, the analytical plan, interpretation of the results, and drafted the manuscript. BZ participated in study design, the analytical plan, statistical analyses, interpretation of the results, and the drafting of the manuscript. JK participated in the design of the analytical plan, interpretation of the results, and the drafting of the manuscript. CC participated in the design of the analytical plan, interpretation of the results, and the drafting of the manuscript.

## Pre-publication history

The pre-publication history for this paper can be accessed here:


